# Therapeutic properties of plant-derived prebiotics in melanoma

**DOI:** 10.37349/etat.2025.1002354

**Published:** 2025-12-19

**Authors:** Emily Kay, Mahnaz Kazi, Jeremy Burton, Seema Nair Parvathy

**Affiliations:** IRCCS Istituto Romagnolo per lo Studio dei Tumori (IRST) “Dino Amadori”, Italy; ^1^Dept of Medicine, Infectious Diseases Division, Western University, London, ON N6A 3K7, Canada; ^2^Lawson Research Institute, London, ON N6A 4V2, Canada; ^3^Dept of Microbiology and Immunology, Western University, London, ON N6A 3K7, Canada

**Keywords:** prebiotics, melanoma, gut microbiome, immunotherapy, inulin, beneficial bacteria

## Abstract

Immune checkpoint inhibitor (ICI) therapy has revolutionized metastatic melanoma treatment, yet only a subset of patients respond effectively, and the treatment can induce a variety of immune-related adverse events (irAEs), including colitis. The gut microbiome plays a critical role in determining patient responses to immunotherapy, prompting exploration of gut-modifying strategies such as prebiotics, probiotics, and fecal microbiota transplantation (FMT) to overcome both primary and acquired resistance and improve treatment outcomes. Prebiotics, defined as dietary substrates that selectively support the growth and/or activity of beneficial gut microorganisms, represent a feasible and safe strategy for microbiome reshaping. Plant-derived prebiotics like castalagin, inulin, fructooligosaccharides, galactooligosaccharides, mushroom extract, kale extract, and konjac glucomannan offer unique advantages over synthetic or animal-derived alternatives due to their natural fiber content alongside their ability to enhance gut microbial diversity. Prebiotics are known to achieve health benefits by selectively stimulating beneficial gut bacteria, producing short-chain fatty acids (SCFAs) that modulate the host immune system, suppressing pathogenic microbes, enhancing mucin production, and modulating systemic and gut-associated immune responses. SCFAs generated through prebiotic fermentation influence host innate and adaptive immunity and regulate metabolic activity via inhibition of histone deacetylases (HDACs), influencing mTOR/MAPK signaling and cytokine production. They also act as ligands for G-protein-coupled receptors (GPCRs), altering intracellular calcium and cAMP to modulate immune cell gene expression. However, the specific mechanisms by which individual prebiotics interact with host genetics, beneficial gut bacteria, and their metabolites are not very well understood. This is crucial to optimize their therapeutic potential in cancer immunotherapy. This review synthesizes current evidence on plant-derived prebiotics, highlighting the impact of beneficial gut bacteria and their metabolites. Given their established safety for human consumption, prebiotics represent a promising, low-risk option to improve gut microbiome composition and potentially enhance immunotherapy and clinical outcomes in cancer.

## Introduction

The gut microbiome is a complex and dynamic ecosystem that has coevolved with the human host, adapting over time in response to physiological, dietary, and environmental factors [1]. From birth, it is shaped by factors such as diet, genetics, and environment, ultimately becoming a key determinant of overall health. Intestinal bacteria and their metabolites support host well-being by providing essential nutrients, enhancing resistance to pathogens, regulating epithelial cell turnover, and modulating immune response [2]. Hence, any shift in the microbiota results in disturbance of this homeostatic mechanism and may promote a diseased state termed “dysbiosis” [3, 4].

Within this microbial landscape, prebiotics have emerged as promising modulators, which are non-digestible dietary ingredients that selectively stimulate beneficial microorganisms [5]. Evidence from multiple studies indicates that both high fiber diets and prebiotic supplementation improve progression-free survival in melanoma patients [6–9]. Beyond gut health, prebiotics may play a role in cancer management by reshaping the microbiota in ways that influence immune responses and therapeutic efficacy [10]. A growing body of clinical research now explores whether plant-based prebiotics can favourably alter melanoma outcomes, bridging the gap between preclinical findings and human studies. Plant-derived prebiotics represent an accessible, culturally familiar, and low-cost intervention, in contrast to engineered oligosaccharides [e.g., 2’-FL, LNnT, synthetic galactooligosaccharides (GOS)], which require industrial fermentation and remain cost-restrictive in many settings. Their natural occurrence in widely consumed foods also facilitates dietary translation and equitable implementation in cancer care.

Given this growing body of evidence, it is important to first summarize the biology of melanoma, an aggressive form of skin cancer that arises from melanocytes, the pigment-producing cells within the epidermis. It is primarily driven by exposure to ultraviolet radiation (UVR), whether from natural sunlight or artificial sources such as tanning beds [11]. Although melanoma represents a relatively small fraction of total global cancer diagnoses, it accounts for most skin cancer-related deaths, underscoring its lethality and need for improved therapeutic strategies [12]. Moreover, there have been extensive studies showing the involvement of the gut microbes in melanoma patients [13]. A significant disruption in the gut microbiota of melanoma patients was observed as compared to controls. The melanoma patients were presented with an abundance of *Fusobacterium*, yeasts belonging to the order of *Saccharomycetales* and *Prevotella copri* species, and a decreased abundance of Actinomycetota and Firmicutes phyla members [14, 15]. The authors also showed that the early-stage melanoma patients had a higher alpha diversity and a higher abundance of the genus *Roseburia* as compared to metastatic melanoma patients, confirming microbiota alteration in the diseased condition.

## Prebiotics

Modulation of the gut microbiota using prebiotics, probiotics, and fecal microbiota transplantation (FMT) has emerged as a complementary strategy to improve immunotherapy outcomes and address treatment resistance in melanoma [16]. Prebiotics, defined as dietary substrates that selectively support the growth and/or activity of beneficial gut microorganisms, represent a feasible and safe strategy for microbiome reshaping. Several natural prebiotics, such as resistant starch, pectin, inulin, and ginseng polysaccharides, have been widely investigated, yet their exact mechanisms in promoting beneficial gut bacteria are still not fully understood [2, 17, 18].

However, the effectiveness of a prebiotic also depends on the presence and abundance of target bacterial populations within the host gut. Factors such as aging and antibiotic usage can lower these bacterial levels, underscoring the importance of evaluating prebiotic efficacy across diverse clinical populations [2]. For instance, resistant starch has been shown to enrich gut bacteria such as *Ruminococcus* and *Bifidobacterium*, which are both associated with beneficial effects on intestinal inflammation, including DSS-induced colitis and enhanced antitumor activity of immune checkpoint inhibitors (ICIs) in mouse models [19].

### Role of prebiotics in melanoma

Emerging evidence suggests that prebiotics may support melanoma therapy as they have the capacity to modulate the gut microbiome and metabolome. To understand the potential of prebiotics in melanoma, it is necessary to review the studies conducted across various models, ranging from in vitro systems to animal studies and clinical trials. Recent findings suggest that a high-fiber, prebiotic-rich diet enhances progression-free survival and immunotherapy response in melanoma patients, likely by promoting beneficial gut microbiota. In contrast, the use of certain commercial probiotic supplements, often consumed for their anti-inflammatory properties in health conditions, has been associated with impaired immune responses and reduced treatment efficacy, highlighting the importance of supporting the native healthy microbiome rather than introducing specific external strains during cancer therapy, as this may lead to further complications [20]. Moreover, the authors have shown probiotic use to be associated with impaired response to immune checkpoint blockade, larger tumors, lower gut microbiome diversity, and fewer cytotoxic T cells in the tumor microenvironment. A possible reason for impaired immunity could be that probiotic use disrupts the gut microbial diversity. Spencer et al. [8] have shown that microbial alpha diversity and *Ruminococcaceae* family and *Faecalibacterium* genus abundances were higher in patients without probiotic use, and that the microbiome and antitumor immunity were impaired in mice receiving probiotics. FMT, in contrast, is a holistic intervention that introduces the stool donors’ entire stool ecosystem for restoration of the recipient gut microbiome rather than specifically targeting bacterial strains. However, variable efficacy of donor stool, unclear mechanistic pathways, and potential transmission of pathogens or opportunistic microbes, and the need to assess safety in a disease-specific context, limit its application [21].

Given these limitations, prebiotics are particularly important to investigate further as they offer a promising alternative to probiotics and FMT. Furthermore, mechanistic studies reveal that combining probiotics with immunotherapy impairs T-helper cell activity, reducing antigen presentation and decreasing dendritic cell function in mouse models, ultimately limiting immune responses and microbial diversity [22]. This reinforces the potential value of prebiotics, which can strengthen immune modulation through endogenous microbial pathways rather than external supplementation and unclear mechanistic pathways for FMT.

### Mechanisms of action of prebiotics

**Figure 1:**
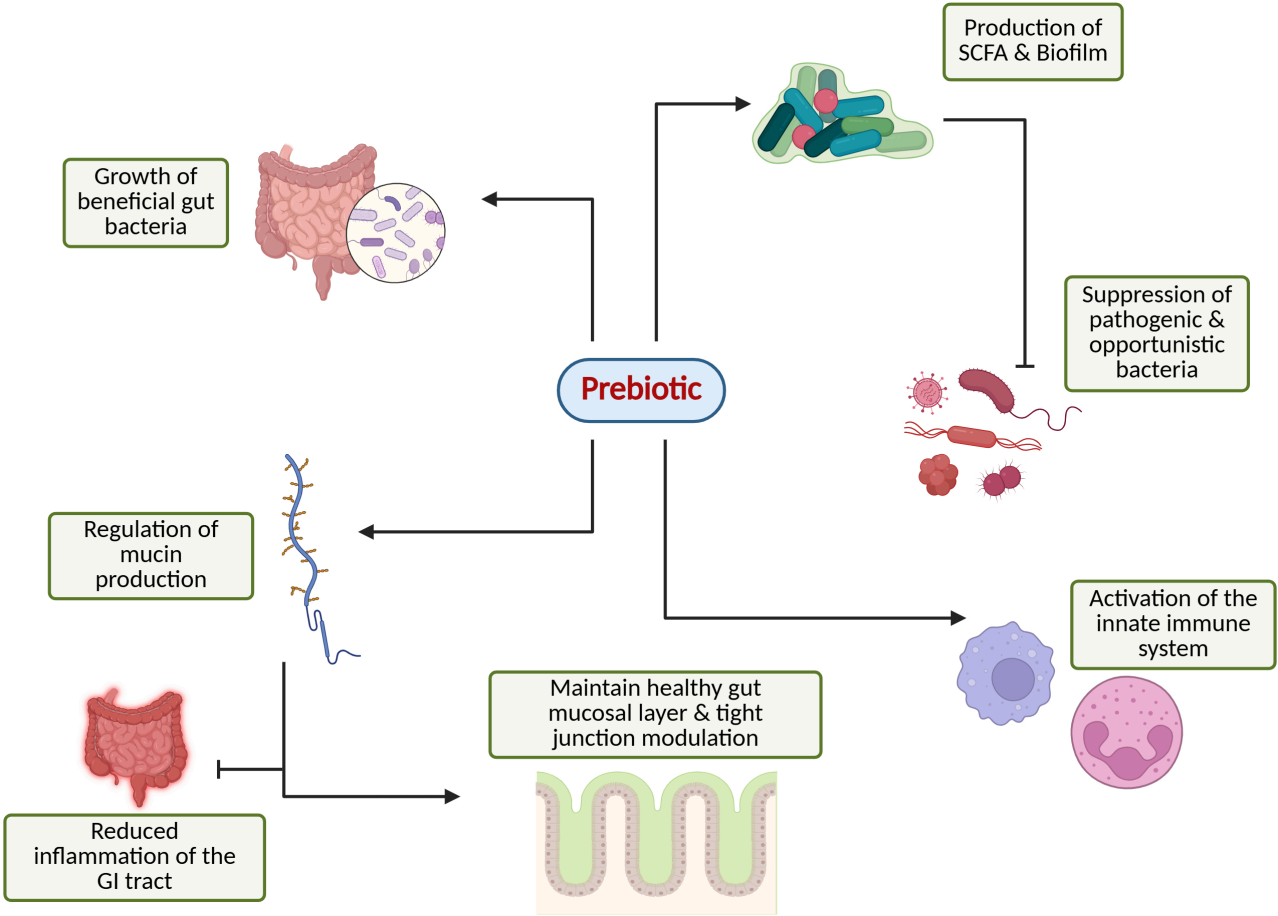
**Overview of the prebiotic potential in modulating gut and immune system function.** Created in BioRender. Parvathy, S. (2025) https://BioRender.com/rfh1d8k.

As illustrated in Figure 1, prebiotics achieve health benefits by exerting their effects through several key mechanisms, including the following:

#### Selective stimulation of beneficial gut bacteria

Prebiotics function as fermentable substrates, serving as nutritional sources for target microbes and promoting their metabolic activity. Whereas simple mono- and disaccharides are swiftly absorbed by the small-intestinal epithelium and thus seldom reach the distal small intestine or colon, fermentable prebiotics resist host digestion and absorption in the upper gut, thereby delivering primary carbohydrate substrates to the large-intestinal microbiota. This provision of saccharolytic fuel helps steer microbial metabolism away from proteolytic and putrefactive pathways, and supports beneficial fermentative activity [e.g., short-chain fatty acid (SCFA) production] by resident microbes. This activity is associated with multiple physiological benefits, including enhanced gut homeostasis, improved nutrient absorption, and the production of health-promoting compounds. Additionally, prebiotics contribute to appetite regulation and may help combat antimicrobial resistance by enriching immune-supportive bacterial species [18, 23].

#### SCFAs and immune pathways

**Figure 2:**
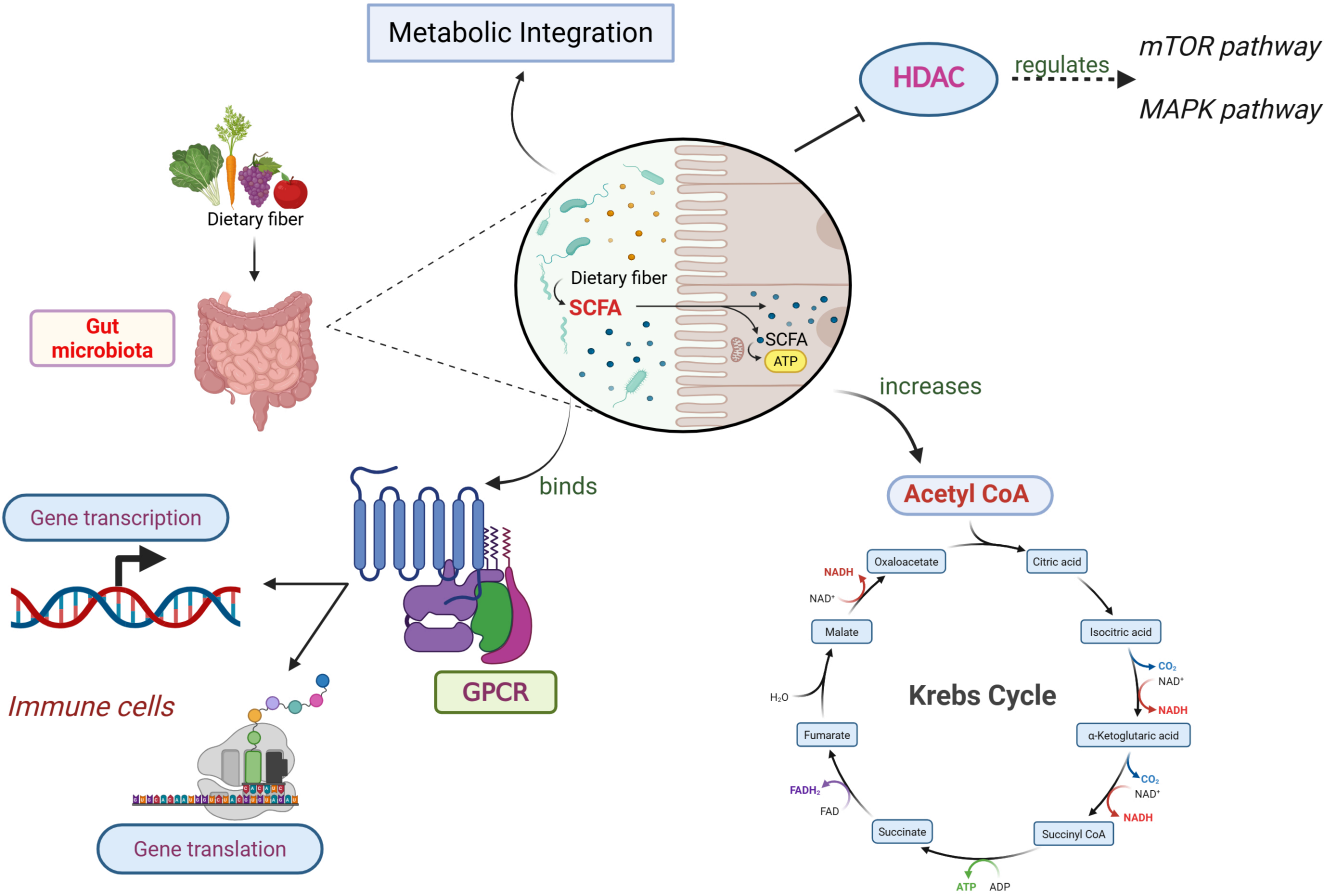
**Downstream effects of SCFAs produced by gut microbiota.** SCFAs regulate immune responses through multiple pathways. They inhibit histone deacetylases (HDACs), influencing mTOR/MAPK signalling and cytokine production, and act as ligands for G-protein-coupled receptors (GPCRs), altering intracellular calcium and cAMP to modulate immune cell gene expression. SCFAs also contribute to cellular metabolism by increasing acetyl-CoA availability. Depending on the context and cell type, SCFAs can exert anti-inflammatory or pro-inflammatory effects. Together, these mechanisms link microbial fermentation of dietary prebiotics with host immune regulation. SCFAs: short-chain fatty acids. Created in BioRender. Parvathy, S. (2025) https://BioRender.com/vlza4p9.

SCFAs, such as acetate, propionate, and butyrate, are key metabolites generated through prebiotic fermentation by gut microbiota that influence host innate and adaptive immunity and metabolic regulation. As shown in Figure 2, SCFAs act through a variety of mechanisms, exerting a downstream effect on the immune system. Chiefly, SCFAs inhibit the histone deacetylases (HDACs), consequently regulating mTOR and MAPK pathways, which are involved in chemokine and cytokine production [24, 25]. Second, SCFA binds to the G-protein-coupled receptors (GPCRs) leading to cascade of downstream effects. The entry of intracellular calcium ions into the cytoplasm increases due to reduced intracellular cAMP levels, thereby regulating the gene transcription and translation in immune cells [26]. This initiates anti-inflammatory signaling cascades and strengthens gut barrier integrity [27, 28]. SCFAs also act via increasing acetyl-CoA production and metabolic integration [29]. In fact, SCFAs are shown to have proinflammatory effects as well as anti-inflammatory effects on different cell types, such as macrophages and microglia [30, 31]. These mechanisms collectively bridge microbial metabolism with immune modulation, providing a mechanistic basis for how prebiotic-derived metabolites can enhance antitumor immunity in melanoma [32].

#### Suppression of pathogenic or opportunistic microbes

By favoring commensal microbial growth, prebiotics can indirectly inhibit harmful bacteria or control pathobionts through competitive exclusion for nutrients and attachment sites, as well as through the production of antimicrobial metabolites like SCFAs, which create an inhospitable environment for harmful pathogens [18, 33].

#### Enhancement of mucin production and mucosal barrier function

Prebiotics promote mucin synthesis and support mucin-degrading bacteria, helping maintain a well-balanced mucus layer. A robust mucosal barrier is critical in protecting the gut epithelium from pathogens, toxins, and inflammation, while tight-junction assembly further strengthens the barrier [33, 34].

#### Modulating systemic and gut-associated immune responses

**Figure 3:**
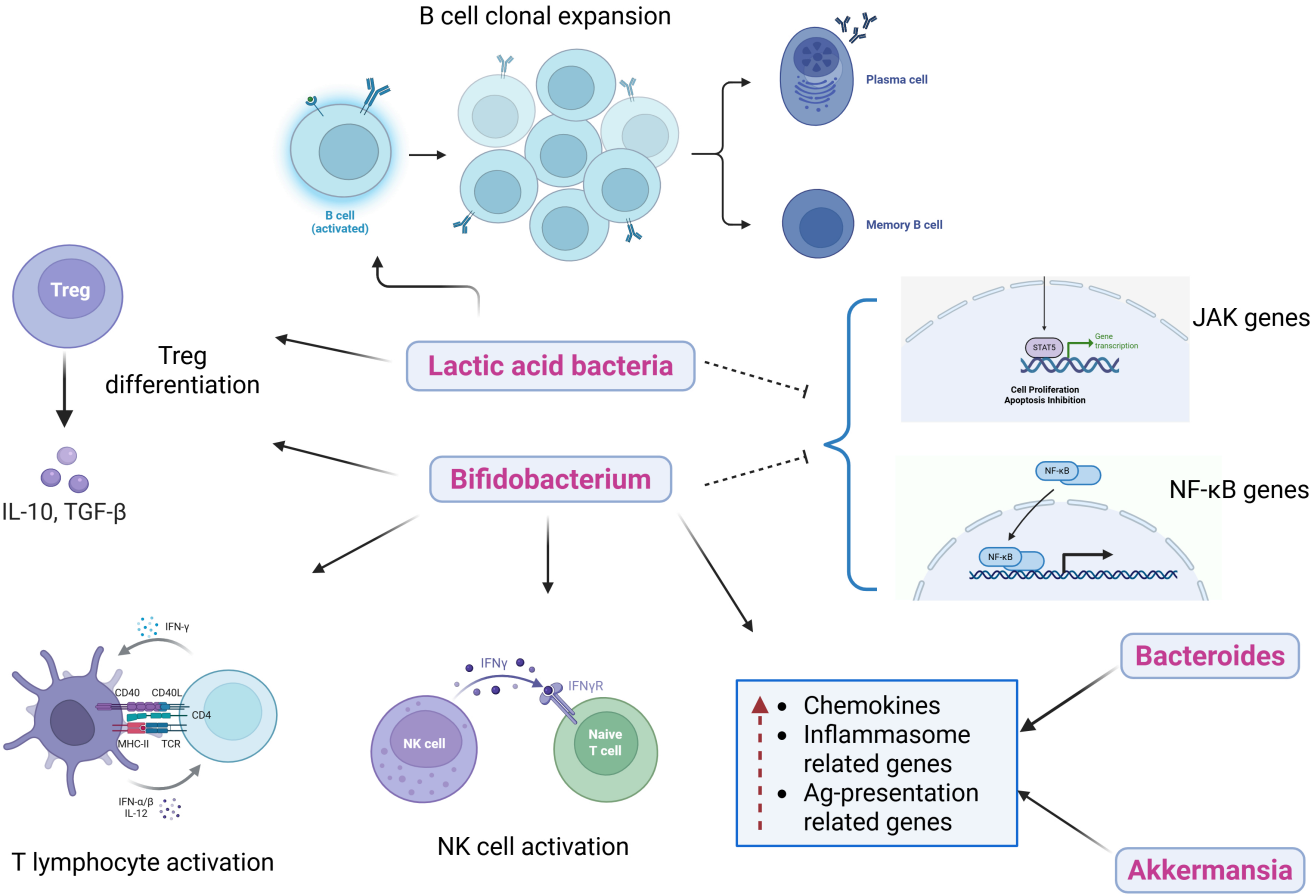
**Effects of different gut microbiota on innate and adaptive immunity.** Prebiotics promote the growth of beneficial gut bacteria that influence immune signalling pathways. These microbes stimulate anti-inflammatory responses through GPCR and pattern-recognition receptor activation, supporting dendritic cell maturation and the recruitment of macrophages, lymphocytes, and other innate immune cells. Beneficial taxa enhance T regulatory and effector T cell activity, increase IgA production, and improve antigen presentation within the tumour microenvironment. Together, these interactions strengthen immune surveillance and may enhance antitumor responses to therapies such as PD-1 blockade. GPCRs: G-protein-coupled receptors. Created in BioRender. Parvathy, S. (2025) https://BioRender.com/qed8cnw.

Prebiotics enhance gut microbiota that influences innate and adaptive immunity through various mechanisms, as can be demonstrated from Figure 3. The anti-inflammatory response is stimulated through pattern recognition receptors or GPCRs, promoting the increased production of inflammatory regulators, including dendritic cells, lymphocytes, monocytes, macrophages, and various innate immune factors, thereby enhancing immune surveillance and strengthening disease resistance [35]. Different genera of lactic acid bacteria (*Lactobacillus casei*, *L. acidophilus*, *L. rhamnosus*, *L. delbrueckii* subsp. *bulgaricus*, *L. plantarum*, *L. lactis*, and *Streptococcus thermophilus*) have immunomodulatory effects through various approaches. They have been shown to promote IL-6 release, which stimulates B cell clonal expansion leading to IgA release [36, 37]. Also, *Bifidobacterium* and *Lactobacillus* stimulate differentiation of T regulatory cells (Treg cells), inhibit the expression of *JAK* and *NF-κB* genes, increase the total helper T cells (CD4^+^) and activate T lymphocytes (CD25^+^) and natural killer (NK) cells [38, 39]. Li et al. [40] found that *Bifidobacteria*, *Bacteroides*, and *Akkermansia muciniphila* lead to an elevated expression of chemokines (CCL4 and CCL8), inflammasome-related genes (*TLR3* and *TLR7*), and antigen presentation-related genes (*CD40*, *Stat1*, and *ICOS*). This indicates a mechanism by which the beneficial gut bacteria enhanced the recruitment and activation of immune cells in the tumor microenvironment, thereby imparting anti-tumor immunity. Furthermore, *Akkermansia*, *Bifidobacterium*, and *Faecalibacterium* are shown to be associated with programmed death 1 (PD-1) blockade in cancer patients, which is usually expressed by tumor cells to prevent the immune system response and suppress anti-tumor immunity [41–44].

## Plant-derived prebiotics against melanoma

Plant-based prebiotic compounds that may provide therapeutic benefits in melanoma are listed below (Table 1).

**Table 1:** Summary of plant-derived prebiotics and their effects on the growth of beneficial gut microbiota contributing to melanoma immunomodulation.

**Prebiotic**	**Plant sources**	**Beneficial bacteria stimulated**	**Immune response/prebiotic effect**	**Source**
Castalagin	Camu camu berry (*Myrciaria dubia*)	*Alistipes*, *Bifidobacterium* spp., *Ruminococcaceae*	Boosts CD8^+^, CD4^+^, and FOXP3^+^ T cell responses Enhances anti-PD-1 immunotherapy efficacy in melanoma	[45, 46, 48, 49]
Inulin	Chicory (*Cichorium intybus*), yacon (*Polymnia sonchifolia*), dried chamomile tea (*C. recutita*), dahlia (*Dahlia pinnata*), Jerusalem artichoke (*Helianthus tuberosus*)	*Bifidobacterium adolescentis*, *Bifidobacterium longum*, *Bacteroides stercoris*, *Faecalibacterium prausnitzii*, *Lactobacillus* spp.	Inhibits *BRAF*-mutant melanoma growth Enhances MEK inhibitor efficacy Protects the colon from LPS-induced oxidative damage and reduces harmful bacteria through SCFA production	[1, 40, 50, 51]
Galactooligosaccharides (GOS)	Non-fructosylated α-GOS purified from peas (*Pisum sativum*)	*Bifidobacterium adolescentis*, *Bacteroides thetaiotaomicron*, *Lactobacillus* spp.	Induces mucin-glycan pathways Enhances SCFA production, strengthens the gut barrier, and boosts immune responses	[35, 40, 52–55]
Kale extract (KE) and lyophilized mushroom extract (LME)	KE from *Brassica oleracea* and LME from *Pleurotus ostreatus*	*Akkermansia muciniphila*, *Bacteroides dorei*, *Bacteroides massiliensis*, *Bacteroides ovatus*, *Blautia producta*, *Blautia producta ASV*, *Bacteroides thetaiotaomicron*	SCFA production (succinic, butyric acids specifically)	[63–65]
Konjac glucomannan (KGM)	*Amorphophallus konjac* (Konjac root vegetable)	*Bifidobacterium adolescentis*, *Lactobacillus acidophilus*, *Lactobacillus casei*	Enhances fermentability, boosting SCFA production Increases lymphocyte proliferation, NK cell activity, and cytokine secretion	[68–70]
Fructooligosaccharides (FOS)	Soybean, roots of yacon (*Polymnia sonchifolia*), and dried chamomile tea (*C. recutita*)	*Lachnospiraceae* spp., *Lactobacillus* spp.	Modulates metabolism Suppresses inflammation Strengthens tight junctions Enhances immune signaling through increased intracellular Ca^2+^ and kinase activity	[34, 35, 58, 59]

SCFA: short-chain fatty acid; NK: natural killer.

### Castalagin

Castalagin is a naturally occurring polyphenol that demonstrates promising prebiotic properties by promoting the growth of immunostimulatory gut bacteria [45]. This microbial modulation enhances immune activation by increasing key immune cell populations such as CD8^+^, CD4^+^, and FOXP3^+^ T-cell populations, which are critical for effective anti-tumor immunity. Notably, castalagin has been shown to enhance the efficacy of anti-PD-1 immunotherapy and may help overcome treatment resistance as observed in melanoma models [46]. These findings highlight castalagin’s potential as a natural prebiotic that not only supports gut health but also contributes to immune-mediated melanoma control.

Oral supplementation of camu camu (*Myrciaria dubia*), a polyphenol-rich berry containing castalagin, has demonstrated the ability to beneficially alter gut microbial composition, increasing the abundance of bacterial taxa associated with improved immunotherapeutic responses in melanoma [45]. In both mice and humans, castalagin supplementation induced a shift in the gut microbiota towards an increase in beneficial bacteria such as *Ruminococcaceae* and *Alistipes*, which are linked to favourable ICI outcomes [47].

Castalagin’s antitumour activity appears to result from the modulation of gut microbiota, enhancing the efficacy of anti-PD-1 antibodies even in antibiotic-treated mice reconstituted with fecal microbiota from ICI-resistant melanoma patients. To enhance the stability and bioavailability, camu camu is often processed through freeze-drying or spray-drying, which allows for its incorporation into foods such as yogurt, grape juice, candies, and bakery products [48]. Notably, camu camu seeds and peels, which are typically discarded as waste products, contain the highest concentrations of polyphenols (182.56 ± 1.76 mg/100 mL), offering an opportunity for sustainable repurposing within the camu camu industry [49].

However, despite its demonstrated immunomodulatory potential, the optimal dosage and human bioavailability of castalagin remain largely undefined. Current evidence is limited to preclinical and small translational studies, with no standardized intake guidelines or pharmaceutical knowledge available. Given castalagin’s polyphenolic nature and susceptibility to extensive first-pass metabolism, factors such as food matrix composition and formulation method are likely to influence its bioavailability and therapeutic potency. Further research optimizing delivery formulations and assessing variability will be critical to advance castalagin toward clinical application.

### Inulin

Inulin shows significant promise in the context of melanoma by enriching antitumor microbial populations and enhancing the therapeutic efficacy of MEK inhibitors, particularly in *BRAF*-mutant melanoma, a subtype characterized by aggressive cell growth and poor prognosis [1, 40]. SCFAs produced during inulin fermentation play a pivotal role in maintaining immune tolerance and preventing excessive inflammatory responses. These metabolites modulate immune homeostasis and enhance targeted antitumor activity [1].

Additionally, molecular dihydrogen (H_2_) produced during inulin fermentation can diffuse across the intestinal barrier, contributing to immune regulation by increasing CD4^+^ and CD8^+^ T-cell activity and inhibiting melanoma tumor growth [50].

Natural sources of inulin are the roots of chicory (*Cichorium intybus*) and yacon (*Polymnia sonchifolia*), as well as the tuber of dahlia (*Dahlia pinnata*) and Jerusalem artichoke (*Helianthus tuberosus*). Inulin with a higher degree of polymerization resists digestion longer, reaching the lower colon where it is fermented by gut microbes [51]. Regular intake of inulin (approximately 5 g per serving, three times daily) can significantly reshape gut microbial diversity within weeks, though research continues to focus on minimizing gastrointestinal discomfort at higher doses [51].

### Galactooligosaccharides (GOS)

GOS exerts its prebiotic effects by activating mucin-glycan pathways and providing β-(1→4)-galactan as a nutrient source for beneficial bacteria. This supports microbial growth, enhances gut barrier integrity, promotes SCFA production, and strengthens mucosal immunity [52]. In cancer models, GOS-induced mucin activation has been linked to antitumor immune responses, particularly through increased efficacy of MEK inhibitors and delayed resistance. These effects are supported by upregulation of MHC class I/II molecules, chemokines (CCL4, CCL8), and immune-activating genes (*CD40, Stat1, ICOS*) [40]. β-GOS is commonly added to dairy products such as infant formula and yoghurt to enhance prebiotic content and support gut health, and is produced by industrial processes of modifying lactose [53]. However, non-fructosylated α-GOS derived from peas (*Pisum sativum*) represents a promising plant-based alternative, offering similar benefits with fewer gastrointestinal side effects, particularly by reduced gas production during colonic fermentation [54, 55]. Preclinical studies have reported beneficial effects at doses of approximately 5 g/kg body weight in animal models [56], while recent human supplementation trials indicate that GOS can exhibit prebiotic efficacy at doses as low as 1.3–2.0 g/day in healthy adults [57]. However, tolerance and clinical outcomes vary among individuals, as higher intakes may cause gastrointestinal discomfort, and differences in gut microbial composition that can alter metabolic response.

### Fructooligosaccharides (FOS)

FOS are typically of shorter chain length than related sugars, such as inulin. FOS contributes to intestinal health and immune regulation, making it a promising adjunct for enhancing anti-melanoma therapies. By modulating cellular metabolism, reducing inflammation, and reinforcing tight junctions within the intestinal epithelium, FOS maintains gut integrity and supports immune activity [34]. Kinome analyses and calcium signaling studies suggest that FOS may boost kinase activity and intracellular calcium levels, which are essential for immune cell activation and antitumor responses [58].

Soybeans are a rich natural source of FOS, highlighting the potential of plant-based foods in promoting gut and immune health. FOS have also been incorporated into soy-kombucha fermentations to produce nutrient-enhanced, dairy-free alternatives with improved flavour and reduced environmental impact compared to animal-based dairy products [59]. Additionally, chamomile tea has been identified as a rich natural source of FOS, containing up to 20% of FOS by dry leaf weight [60], and the roots of *Polymnia sonchifolia* (yacon) provide another sustainable source for functional food applications [61].

While FOS exhibits prebiotic, gut-barrier, and immune-supporting activities, standardized human dosing and formulation bioavailability remain ongoing. Clinical studies have shown that supplementation at doses of approximately 7.5–15 g/day for four weeks or longer significantly increases *Bifidobacterium* abundance compared to lower doses [62]. Additionally, phase I trials have also demonstrated that FOS is well tolerated at up to 10 g/day, with only mild gastrointestinal symptoms reported [62].

### Lyophilized mushroom extract (LME) and kale extract (KE)

Mushrooms, particularly *Pleurotus ostreatus*, serve as rich natural sources of prebiotics due to their bioactive polysaccharides like chitin, hemicellulose, α- and β-glucans, mannans, xylans, and galactans. These compounds resist acid hydrolysis and digestion in the upper gastrointestinal tract, which enables LME to promote SCFA production and beneficial bacterial growth, mirroring similar effects observed with inulin [63, 64].

When combined with KE, these natural prebiotics synergistically enhance the production of SCFAs such as succinic acid and butyric acid, which are compounds that are well known for their immunomodulatory and antitumor properties. KE contributes additional minerals and beneficial prebiotic carbohydrates such as FOS, amplifying the overall gut-supportive and immune-enhancing activity [65].

Human trials report immune-stimulatory effects at oral doses of 100–500 mg/day of β-glucans derived from LME, though absorption and efficacy are variable as they depend on molecular weight and branching structure [66]. These findings underscore its prebiotic and immunological potential in the context of melanoma. Human dosage data for KE in prebiotic and immunomodulatory applications remain unavailable, as most findings are derived from pre-clinical or dietary studies. Therefore, further investigations into formulation, bioavailability, and dose-response optimization are needed to confirm the synergistic effects of KE and LME in clinical settings. Overall, these extracts and their biological mechanisms demonstrate strong potential to reinforce gut-mediated immune responses relevant to melanoma therapy [67].

### Konjac glucomannan (KGM)

Hydrolyzed KGM, particularly in the form of KGM oligosaccharides (KGOS), demonstrates more substantial prebiotic effects compared to its unhydrolyzed counterpart. Produced through enzymatic hydrolysis using *Bacillus amyloliquefaciens* WX-1 [68], KGOS is more readily fermented by anaerobic gut bacteria, generating SCFAs that promote gut and immune health. These SCFAs have been shown to improve spleen and thymus function, stimulate T and B lymphocyte proliferation, elevate serum hemolysin levels, increase phagocytic and NK cell activity, along with enhancing the secretion of immune-regulatory cytokines such as TNF-α, IL-2, and IgG [69]. Given melanoma’s reliance on immune activation for effective therapy, KGOS’s ability to activate systemic immunity underscores its therapeutic potential.

KGM is extracted from the tubers of *Amorphophallus konjac*, a plant valued for its high polysaccharide content and established use as a functional food. KGM is extracted through precipitation from dried konjac tubers, which are the primary edible and economically valuable part of the plant [70]. It is composed mainly of glucose and mannose units arranged in long chains. Given the widespread inclusion of probiotics in commercial dairy products, incorporating prebiotic compounds like KGM is a common symbiotic strategy for improving colonic and overall health [68].

According to the European Food Safety Authority, daily intake of approximately 3 g of KGM, typically divided into three 1 g doses, is generally well tolerated, although higher amounts may lead to mild gastrointestinal discomfort [71]. While these findings support its use as a safe, fermentable dietary fibre, clinical studies optimizing dose-response relationships and bioavailability in immunological or oncological contexts remain limited, urging further investigation in this area.

When comparing the prebiotics, notable differences in their mechanisms, levels of supporting evidence, and translational potential are observed. Oligosaccharides such as inulin, GOS, and FOS possess substantial in vivo and clinical results for modulating the gut microbiota and metabolic function, yet their influence on tumor immunity remains indirect. In contrast, castalagin and KGM demonstrate promising in vivo immunomodulatory effects, primarily through SCFA-mediated enhancement of gut-immune signaling. LME and KE further contribute synergistic prebiotic and immunological effects in preclinical studies, though standardized dosing and bioavailability data remain limited. Collectively, the comparison of these compounds underscores that while classical oligosaccharides provide strong clinical safety and efficacy profiles, novel polyphenol and polysaccharide-based prebiotics such as castalagin and KGM represent emerging candidates with distinct structural complexity and target immunomodulatory potential but require further mechanistic and clinical investigation before translation to oncology settings.

## Adverse effects of prebiotics

Although there have been no serious side effects reported to date in cancer patients, some adverse symptoms have been found to be associated with prebiotic consumption. The major adverse effects of prebiotics are diarrhea, bloating, abdominal cramps, and flatulence owing to their osmotic effect [72]. However, the adverse effects are largely dose-dependent, with varying reports by several studies. In fact, different prebiotics have been shown to exhibit different safety profiles of dose for consumption. While low doses (2.5–10 g/day) can cause flatulence due to gaseous fermentation products such as hydrogen gas and carbon dioxide, high doses (40–50 g/day) lead to osmotic diarrhea [73, 74].

## Limitations

Although current evidence highlights the promise of prebiotics in modulating the melanoma-associated microbiome, critical gaps in knowledge remain that require future investigation. As summarized in Table 1, we have limited information regarding natural prebiotic compounds and their effect on beneficial gut bacteria present in responders to melanoma immunotherapy. Future research should therefore focus on identifying or developing prebiotics that selectively stimulate these key microbes and metabolites to optimize therapeutic efficacy.

One major limitation in microbiome-based therapies is the limited understanding of their mechanisms of action and the lack of reliable preclinical models that accurately reflect human responses [47]. These challenges hinder the design of consistent interventions and complicate the translation of promising findings into clinical practice. To address these limitations, in vitro and ex vivo models have been increasingly employed to study how prebiotics influence melanoma metabolism and immune function. While these models offer improved translational relevance, they still cannot fully replicate the complexity of human physiology and the dynamic interactions of the microbiome. While in vitro studies establish mechanistic insights, the dynamic and individualized nature of the human microbiome complicates reproducibility and creates generalizations of results derived from non-human subjects. Similarly, in vivo animal studies often fail to capture the heterogeneity of human tumors, while raising ethical and cost concerns [75]. These limitations collectively slow the progression of preclinical discoveries into clinical trials. Another obstacle lies in the methodological diversity across studies. Variation in microbial classification systems, experimental models, and analytical techniques across both preclinical and clinical settings makes it difficult to directly compare results or draw universal conclusions [76]. Consequently, despite an attempt at a comprehensive review, it may not encompass all relevant literature, underscoring the need for continuous and standardized investigation.

Furthermore, clinical and translational evidence increasingly supports a link between diet, the gut microbiome, and immunotherapy outcomes, while also revealing major challenges in applying these findings to patients. High-fiber diets and probiotic modulation have been shown to influence gut microbial diversity and enhance ICI efficacy in melanoma, but outcomes remain inconsistent across patients, reflecting the complexity of host-microbiome interactions and the influence of individual variability, genetics, and dietary habits [8]. Moreover, translation to clinical applications is also limited owing to methodological gaps, including inconsistent microbial sequencing protocols and the lack of integrative tools to assess host immune responses alongside microbial changes. Recent advances in host-focused molecular technologies have begun to address these issues, offering new approaches to characterize host-microbiome crosstalk with greater precision, yet standardization and reproducibility remain key barriers to implementation [77]. Together, these findings emphasize the need for harmonized clinical frameworks to reliably translate microbiome-based interventions into patient-specific cancer therapies.

Future research must prioritize large-scale, well-controlled clinical trials that validate prebiotic mechanisms in human populations. Host genetics, dietary regimen, and microbiota interactions are crucial to fully understanding the therapeutic potential of plant-based prebiotics. Recognizing these gaps provides a framework for future research, which must focus on systematic evaluation of plant-based prebiotics in melanoma clinical trials that may ultimately lead to precise microbiome-based therapies that complement conventional cancer treatments.

## Conclusions

Prebiotics hold significant promise for supporting gut health and enhancing immunotherapeutic outcomes in melanoma; however, their benefits are not universally applicable. It is essential to avoid overgeneralization, as prebiotic efficacy depends on multiple factors, including microbial composition, host physiology, and disease context. Plant-derived compounds represent an accessible and sustainable approach to therapy, offering natural prebiotic benefits with fewer side effects compared to synthetic alternatives commonly found in commercial products. These natural agents not only contribute to microbial balance but may also augment immune responses and improve patient outcomes. Refining prebiotic formulations to target a broader range of microbial species and immune pathways may pave the way for precision microbiome-based strategies in oncology. Ultimately, integrating plant-based prebiotics into cancer care has the potential to bridge nutrition, microbiology, and immunotherapy, advancing personalized medicine and improving the quality of life for patients with melanoma.

## Abbreviations

FMT: fecal microbiota transplantation
FOS: fructooligosaccharides
GOS: galactooligosaccharides
GPCRs: G-protein-coupled receptors
HDACs: histone deacetylases
ICI: immune checkpoint inhibitor
KE: kale extract
KGM: konjac glucomannan
KGOS: konjac glucomannan oligosaccharides
LME: lyophilized mushroom extract
NK: natural killer
PD-1: programmed death 1
SCFAs: short-chain fatty acids

## Declarations

### Copyright

© The Author(s) 2025.
